# A four-hypoxia-genes-based prognostic signature for oral squamous cell carcinoma

**DOI:** 10.1186/s12903-021-01587-z

**Published:** 2021-05-03

**Authors:** Chenguang Zhao, Yingrui Zhou, Hongwei Ma, Jinhui Wang, Haoliang Guo, Hao Liu

**Affiliations:** 1 Department of Emergency and General Dentistry, Tianjin Stomatology Hospital, School of Medicine, NanKai University, Tianjin Key Laboratory of Oral and Maxillofacial Function Reconstruction, Tianjin, 300041 China; 2 Department of Oral and Maxillofacial Surgery, Tianjin Stomatology Hospital, School of Medicine, NanKai University, Tianjin Key Laboratory of Oral and Maxillofacial Function Reconstruction, No. 75, Dagu North Road, Heping District, Tianjin, 300041 China

**Keywords:** OSCC, Hypoxia, OS, Prognosis

## Abstract

**Background:**

Oral squamous cell carcinoma (OSCC) is one of the most common maligancies of the head and neck. The prognosis was is significantly different among OSCC patients. This study aims to identify new biomarkers to establish a prognostic model to predict the survival of OSCC patients.

**Methods:**

The mRNA expression and corresponding clinical information of OSCC patients were downloaded from The Cancer Genome Atlas and Gene Expression Omnibus. Additionally, a total of 26 hypoxia-related genes were also obtained from a previous study. Univariate Cox regression analysis and LASSO Cox regression analysis were performed to screen the optimal hypoxia-related genes which were associated with the prognosis of OSCC. to establish the predictive model (Risk Score) was established for estimating the patient's overall survival (OS). Multivariate Cox regression analysis was used to determine whether the Risk Score was an independent prognostic factor. Based on all the independent prognostic factors, nomogram was established to predict the OS probability of OSCC patients. The relative proportion of 22 immune cell types in each patient was evaluated by CIBERSORT software.

**Results:**

We determined that a total of four hypoxia-related genes including ALDOA, P4HA1, PGK1 and VEGFA were significantly associated with the prognosis of OSCC patients. The nomogram established based on all the independent factors could reliably predict the long-term OS of OSCC patients. In addition, our resluts indicated that the inferior prognosis of OSCC patients with high Risk Score might be related to the immunosuppressive microenvironments.

**Conclusion:**

This study shows that high expression of hypoxia-related genes including ALDOA, P4HA1, PGK1 and VEGFA is associated with poor prognosis in OSCC patients, and they can be used as potential markers for predicting prognosis in OSCC patients.

**Supplementary Information:**

The online version contains supplementary material available at 10.1186/s12903-021-01587-z.

## Introduction

Oral squamous cell carcinoma (OSCC) is one of the most universal tumors in the head and neck, with the global incidence rate is the eighth of all cancers [1]. Statistically, more than 300,000 new cases are diagnosed worldwide annually [1]. Its pathogenic factors include smoking, drinking, chewing betel nut, carcinogen exposure, viral infection, immunodeficiency, gene specificity, etc. [2, 3]. At present, there were certain advances in surgery, radiotherapy and chemotherapy for OSCC, but the fatality rate remains high [4]. And the survival of OSCC patients is significantly different. Therefore, it is of great significance to screen biomarkers related to prognosis of OSCC.

Many original tumor areas have low concentrations of molecular oxygen, known as hypoxia [5]. Hypoxia is the specific microenvironment of tumor, which is closely associated with the initiation and progression of cancer. The causes include disordered tumor blood vessels leading to insufficient oxygen supply and accelerated tumor metabolism leading to increased oxygen consumption. Hypoxia increases the migration of tumor cells. Hypoxia-inducible factors (HIFs) induce the expression of matrix metalloproteinases and other protease genes, leading to the degradation of the matrix around the tumor and providing channels for tumor metastasis [6]. Hypoxia can change the abundance of gene expression, which will result in proteomics changes and affect cell physiological and biochemical functions [7]. For example, the abundance of DNA double-stranded repair protein RAD51 is down-regulated in anoxic cells, resulting in inhibition of DNA repair. In addition, DNA mismatch repair genes *MLH1* and *MSH2* are down-regulated under hypoxia, leading to a large part of gene mutations [8, 9]. Furthermore, hypoxia affects the sensitivity of tumor cells to chemotherapy and radiotherapy, and its presence has a significant negative impact on the prognosis of patients after radiotherapy [10].

In recent years, the significance of hypoxia in the treatment of cancer has been identified. The earliest research indicated that hypoxia had effect on the proliferation of mouse embryonic fibroblasts, and the inactivation of HIF-1 enhances the inhibition of cell proliferation [11]. Further, overexpression of HIF-1 was found in human tumor tissues commonly, including breast, stomach, skin, ovary, and pancreas [7]. For instance, HIF expression level in pancreatic cancer is significantly increased and can be used as a clinical marker of pancreatic cancer survival rate to assess the prognosis of patients. Platinum insensitivity often occurs during platinum chemotherapy for non-small cell lung cancer (NSCLC), but there is evidence that A549 NSCLC cells are significantly more sensitive to platinum after reoxygenation [12]. However, the prognostic value of hypoxia-related genes is very limited presently, especially in OSCC.

Therefore, in this study, the prognostic value of 26 hypoxia related genes in OSCC was explored, four optimal genes associated with the prognosis of OSCC were identified, and a reliable prognostic model was established, in order to provide some guidance for the clinical application of OSCC.

## Material and methods

### Study subject

The information of 349 OSCC patients about genome-wide expressions and corresponding clinical data were downloaded from The Cancer Genome Atlas (TCGA, https://tcga-data.nci.nih.gov/tcga/), among which 348 patients had complete survival data, which were used for subsequent analysis. In addition, we also obtained mRNA expression and clinical information of 218 OSCC patients with the accession number of GSE65858 from Gene Expression Omnibus (GEO, https://www.ncbi.nlm.nih.gov/geo/) [13]. Clinical information for all patients in the TCGA and GEO datasets is shown in Table1.

**Table 1:** Clinical characteristics of TCGA and GEO sets

Variable	TCGA set (n = 348)	GEO set (n = 218)
Age		
Median	61	58
Range	19–90	35–87
Gender		
Female	106	40
Male	242	178
Clinical T		
T1	19	30
T2	94	65
T3	76	40
T4	106	83
TX	5	0
Clinical N		
N0	160	64
N1	54	30
N2	75	114
N3	3	10
NX	8	0
Clinical M		
M0	288	213
M1	0	5
MX	12	0
TNM stage		
Stage I	20	13
Stage II	55	25
Stage III	61	30
Stage IV	176	150
Grade		
G1	52	N/A
G2	212	N/A
G3	73	N/A
G4	2	N/A
GX	7	N/A
Radiation therapy		
NO	111	N/A
YES	172	N/A
Race		
American Indian or Alaska native	1	N/A
Asian	9	N/A
Black or African American	25	N/A
White	303	N/A
Alcohol History		
NO	112	22
YES	228	196
Lymphovascular invasion		
NO	171	N/A
YES	76	N/A
Perineural invasion		
NO	122	N/A
YES	139	N/A
Vital status		
Alive	191	138
Dead	157	80

N/A, not available

### Hypoxia-related genes

Here the expression value of 26 hypoxia genes previously reported to associate with the efficacy of hypoxia therapy for laryngeal cancer [14] was used to clustered samples, and the 26 hypoxia related genes were shown in Additional file 1: Table S1.

### Cluster analysis

Based on the mRNA expression of the 26 hypoxia-related genes, the function package factoextra (https://cran.cran.project.org/package=factoextra) in R language was used to perform sample clustering analysis.

### LASSO Cox regression analysis

Basing on the mRNA expression values of 26 hypoxia-related genes, the univariate Cox regression analysis was conducted, and the genes significantly related to the prognosis of OSCC were selected with P < 0.05 as the threshold. Then, glmnet package of R language [15] was used for LASSO Cox regression analysis to further refine the hypoxia-related genes related to the prognosis of OSCC, and the Risk Score of each sample was calculated according to the refined genes:

$${\text{Risk Score}} = \mathop \sum \limits_{{{\text{i}} = 1}}^{{\text{n}}} Coef_{i } {*}X_{i}$$

In the formula, *Coef*_*i*_ is the risk coefficient. *X*_*i*_ is the expression value of hypoxia-related genes. The samples were divided into Low Risk group and High Risk group based on the median of Risk Score.

### Survival analysis

The overall survival rate of different groups was estimated based on the Kaplan–Meier method using the survial package and the survminer package of R language, and log-rank test was used to determine the significance of the difference in survival rate between different groups with P < 0.05 as a significance threshold. Finally, multivariate Cox regression analysis was used to determine whether Risk Score was an independent factor to predict the survival of patients with OSCC.

### Analysis of immune cell infiltration

The CIBERSORT software [16] was used to compute the relative ratios of 22 immune cell types in the samples. Based on the gene expression matrix, the deconvolution algorithm used the preset 547 barcode genes to characterize the proportion of the infiltrating immune cells. The sum of the estimated proportions of the 22 immune cells in each sample was 1.

### Immune checkpoints among different groups

The corAndPvalue function in the WGCNA package [17] of R software was used to calculate the correlation between the immune checkpoint and Risk Score. The chordDiagram function in circlize package [18] was conducted for visualization of correlation.

### Construction and evaluation of the nomogram

Based on the independent prognostic factors identified by multivariate Cox regression analysis with RMS (https://cran.cran.r-project.org/package=RMS) package, nomogram was established to predict overall survival probability for 1-year, 3-year and 5-year. A calibration curve was drawn to determine the divergency between Nomogram's predicted probability and actual incidence.

### Statistical analysis

Wilcoxon signed-rank test was performed to compare the expression of immune checkpoints in different groups. P < 0.05 was used as the significance threshold. All statistical analyses were carried out by using R software V3.5.2.

## Results

### Hypoxia-related genes could separate prognostically different OSCC patients

**Fig. 1:**
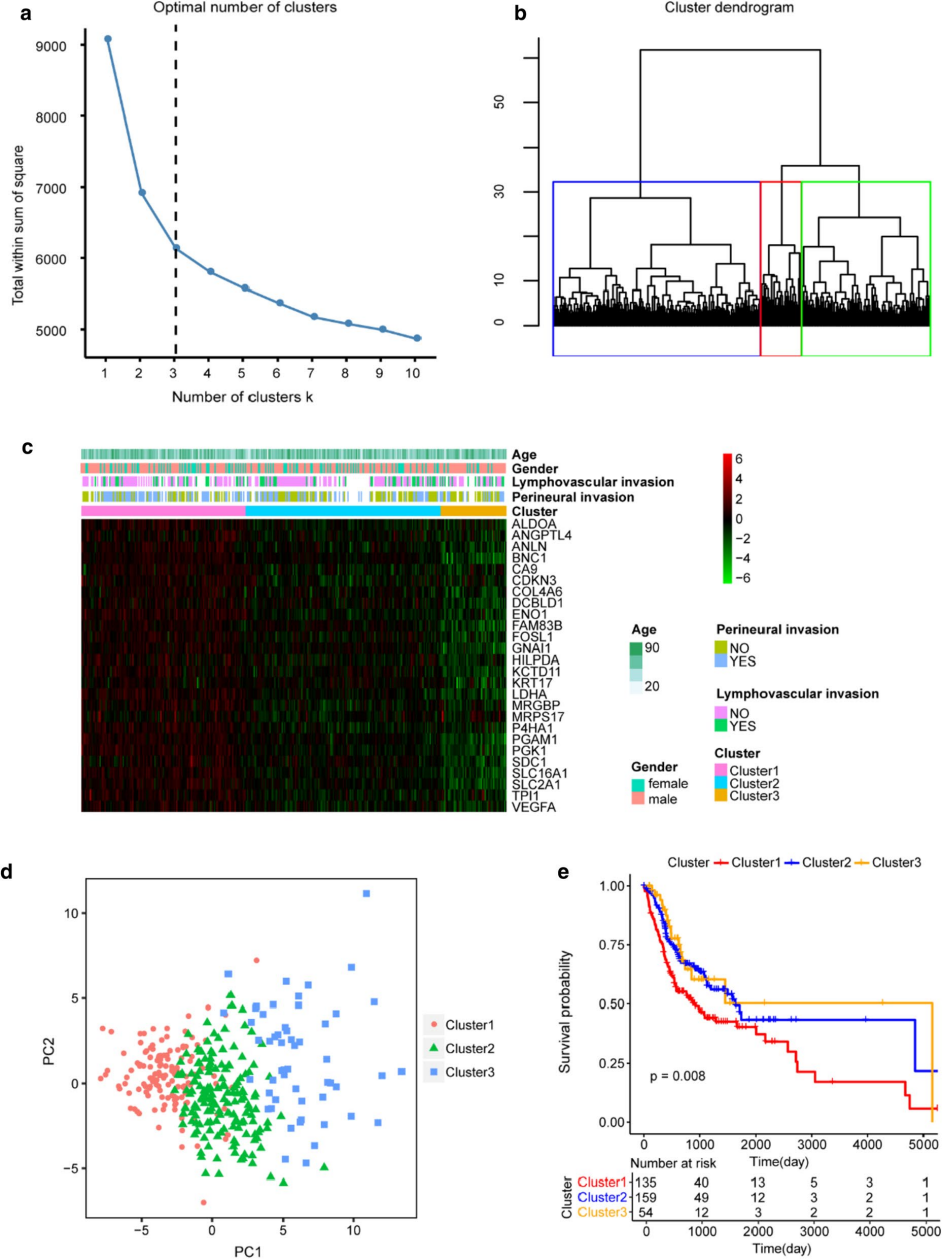
The consensus clustering analysis of OSCC samples in TCGA based on mRNA expression of hypoxia-related genes. **a** Elbow graph that determines the optimal number of clusters. The horizontal axis is the number of clusters K, and the vertical axis is the sum of squared errors (SSE). The point where the decline tends to be gentle is the optimal number of clusters. **b** Cluster dendrogram of OSCC samples. Different colors refer to different clusters. **c** Heat map of the expression of hypoxia-related genes in different clusters. X-axis and Y-axis refers to genes and samples, respectively. The red color represents high expression, and the green color  represents low expression. The age and sex of the sample are marked with different colors above the heat map. **d** The principal component (PCA) analysis. The dots with different colors refer to samples in different groups. The distance of the dots revealed the similarity of the hypoxia-related gene expression. The P value is obtained  from the log-rank test

The factoextra function package in R language was used for clustering analysis for OSCC patients in TCGA based on the expression values of 26 hypoxia-related genes. According to the sum of the squared errors (SSE), the optimal number of clusters was determined as 3 (Fig. 1a). The clustering diagram (Fig. 1b) clearly showed that the OSCC patients from TCGA could classified be basically separated into three clusters, including 135, 159 and 54 cancer samples, respectively. The expression heatmap (Fig. 1c) indicated that the expression levels of Cluster 1 to Cluster 3 were gradually decreased, and the three clusters could be obviously identified. The above result was also supported by principal component analysis (PCA) (Fig. 1d), which revealed the differences of the three clusters of samples were obvious and the samples of the same Cluster were more closely similar. Kaplan–Meier survival analysis showed that there were obvious differences in the overall survival (OS) among the three OSCC groups (P = 0.008) (Fig. 1e), and patients in Cluster 1 had poorer survival. It indicated that hypoxia-related genes could discriminate the prognostically different OSCC samples effectively.

### Hypoxia related genes-based prognostic model for OSCC

**Fig. 2:**
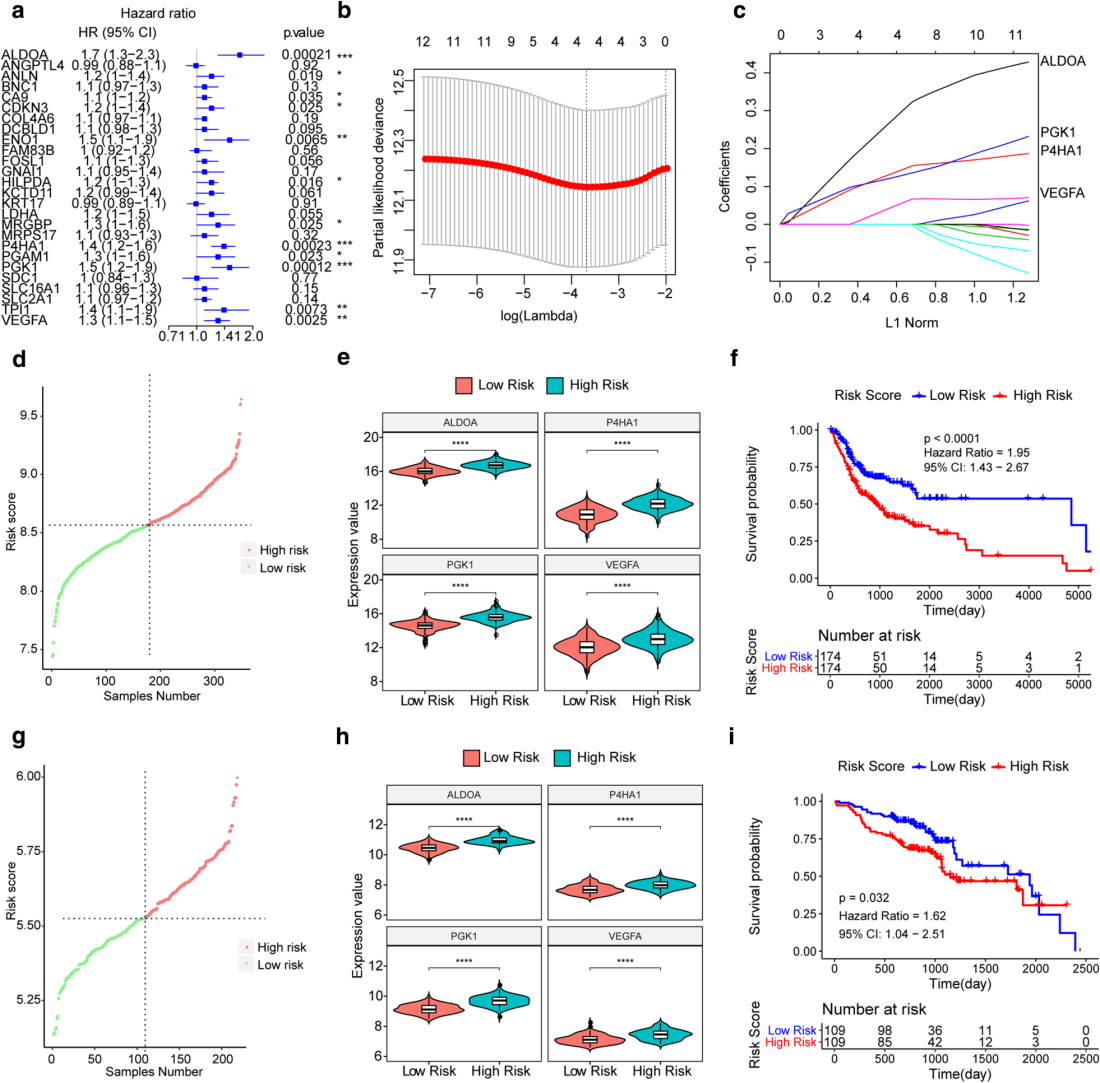
The risk score model could effectively predict the survival of OSCC patients. **a** The forest plot of univariate Cox regression analysis of 26 hypoxia-related genes. HR: Hazard ratio, CI: confidence interval. *P < 0.05. **b** Determination of the tuning parameter lambda by using LASSO Cox regression analysis. The horizontal axis represents log (lambda), and the vertical axis represents partial likelihood deviation. The Lambda value which corresponds to the smallest partial likelihood deviance value is the optimal. That is, the optimal Lambda value after Log is taken below the dotted line, and the corresponding value above is the number of optimal genes. **c** Coefficient spectrum of LASSO Cox regression analysis. **d** The distribution of Risk Scores of the OSCC samples in the TCGA dataset. The red color represents high expression, and the green color represents low expression. **e** Violin diagram of the expression levels of four hypoxia-related genes in the high and low risk groups of TCGA samples. **f** Kaplan Meier survival curve of TCGA samples. The horizontal axis denotes time, the vertical axis denotes survival rate, and different groups are represented by different colors. **g** The distribution of Risk Scores of samples in the GSE65858. **h** Violin diagram of the expression levels of four hypoxia-related genes in the GSE65858 in the high and low risk groups. **i** Kaplan Meier survival curve of GSE65858

The expression values of 26 hypoxia-related genes in TCGA samples were used as continuous variables for univariate cox regression analysis to explore their associations with OSCC patients’ OS probability. There were 12 genes (ALDOA, ANLN, CA9, CDKN3, ENO1, HILPDA, MRGBP, P4HA1, PGAM1, PGK1, TPI1 and VEGFA) determined to be significantly associated with the OS of OSCC (Fig. 2a), which could be all risk genes for OSCC patients (HR > 1, P value < 0.05), indicating that high expression of these genes may cause poor prognosis.

Then the 12 hypoxia-related genes were used for LASSO Cox regression analysis. Based on the lambda value corresponding to the different number of genes, 4 optimal genes (ALDOA, P4HA1, PGK1, and VEGFA) were selected (the lambda value is the smallest) (Fig. 2b, c). Further, we established a predictive risk score model for OS of OSCC patients based on the regression coefficients weighted expressions of the four hypoxia-related genes (Risk Score = 0.2777 * Express Value of ALDOA + 0.1355 * Express Value of P4HA1 + 0.1237 * Express Value of PGK1 + 0.047 * Express Value of VEGFA). The OSCC patients in TCGA set and GEO set were all assigned risk scores based on the model and respectively divided into low- and high-risk groups according to the median of the Risk Score (Fig. 2d, g). It was found that the expression values of hypoxia-related genes in the model were significantly different between the high and low risk groups (Fig. 2e, h). Additionally, the OS of OSCC samples in the high-risk group was poorer than those in the low-risk group in both TCGA and GEO dataset (P < 0.0001 for TCGA, and P = 0.032 for GEO) (Fig. 2f, i). Overall, it showed that the prognostic model constructed using the four hypoxia-related genes, including ALDOA, P4HA1, PGK1, and VEGFA, could well predict the prognosis of OSCC patients.

### Risk Score was significantly associated with OSCC patients’ immune microenvironment

**Fig. 3:**
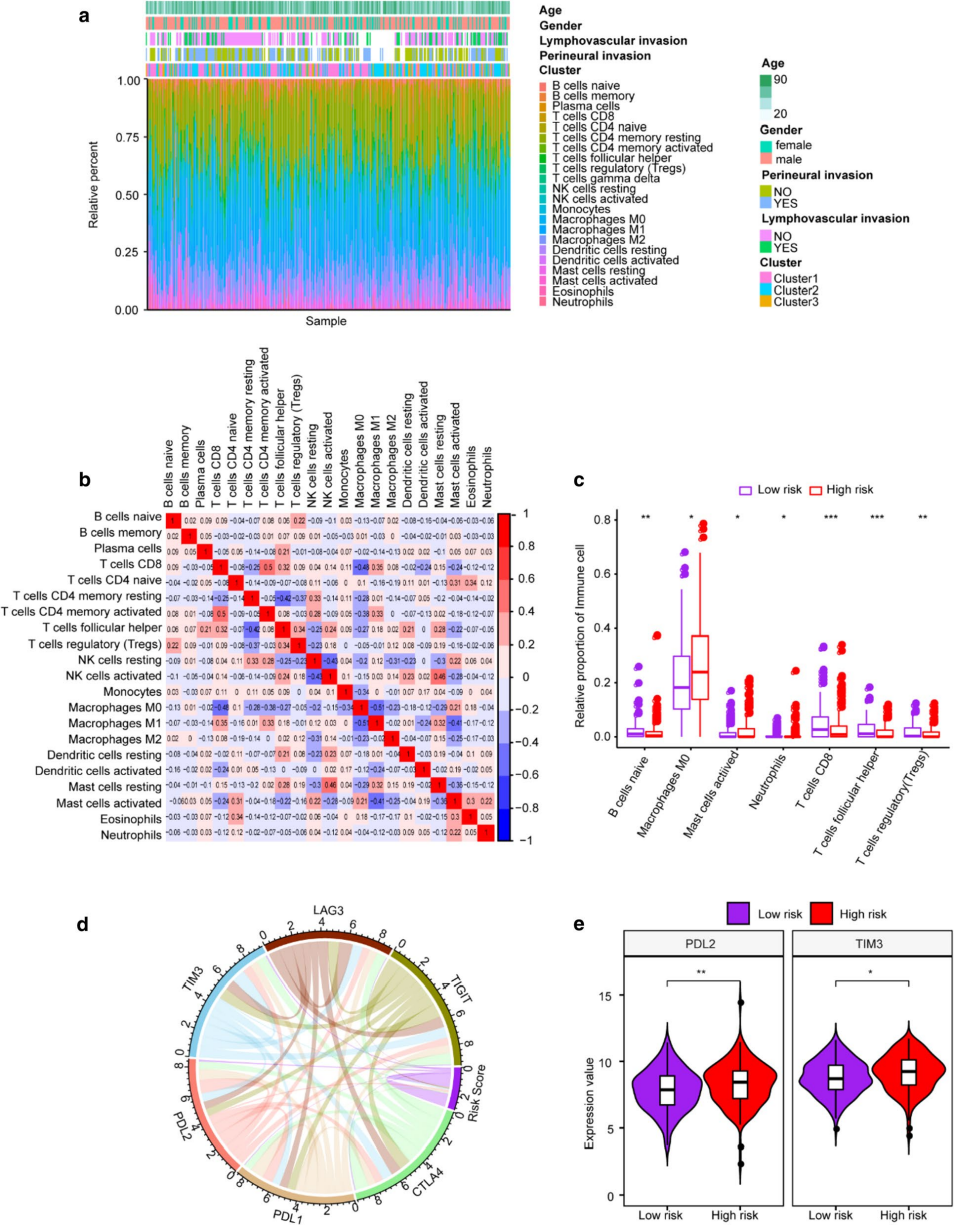
Immune infiltration in OSCC patients with high and low Risk Scores. **a** The infiltration ratio of 22 kinds of immune cells in TCGA samples. **b** Correlation matrix of the ratio of 22 immune infiltrating cells. The red color represents a positive correlation and the blue color represents a negative correlation. **c** Violin diagram of immune cells with significantly different proportions of infiltration in high and low risk groups. The high and low risk groups are represented by different colors , and the vertical axis is the relative infiltration ratio of different immune cells. *P value < 0.05, **P value < 0.01, ***P value < 0.001, and ****P value < 0.0001. **d** The correlation between the Risk Score and the expression of six key immune checkpoints. **e** Differentially expressed immune checkpoints between the high and low risk groups

In this study, CIBERSORT method was used to estimate the relative infiltrating proportions of 22 immune cells in OSCC patients from TCGA. The infiltrating landscape of the 22 immune cells exhibited clear discrepancies among OSCC patients (Fig. 3a). In addition, the correlations between the infiltrating ratios of most different types of immune cells is weak (Fig. 3b), indicating that there was great heterogeneity in the infiltration of different immune cells in OSCC patients. Additionally, it was found that there are 7 types of immune cells that have significant differences in the degree of infiltration between OSCC patients within high- and low-risk groups, including B cells naive, T cells CD8, T cells follicular helper, T cells regulatory (Tregs), Macrophages M0, Mast cells activated and Neutrophils. Moreover, compared to infiltration rates in low-risk group, only Macrophages M0 and Mast cells were significantly higher in the high-risk group, while the other five types of immune cells were markedly lower (Fig. 3c), and difference in the amount of immune cells might be the case for prognostic differences of OSSC patients.

As previously reported, the expression of immune checkpoints has become a biomarker for the selection of immunotherapy [19]. Here we analyzed the correlation between the six immune checkpoints CTLA4, PDL1, PDL2, TIM3, LAG3, TIGIT and the risk score of TCGA samples, and found that the expression of PDL2 and TIM3 was significantly correlated with the Risk Score (Fig. 3d and Additional file 1: Table S2). We also studied the expression of these 6 immune checkpoints in OSCC patients in high and low risk groups, and found that the expressions of PDL2 and TIM3 were significantly different in OSCC patients in high- and low-risk groups, and the high-risk group has higher expression level (P value < 0.05) (Fig. 3e), indicating that the high-risk patients may more sensitive to the treatment of PDL2 and TIM3 immune checkpoint inhibitor.

### Risk Score was an independent prognostic factor for OSCC

**Fig. 4:**
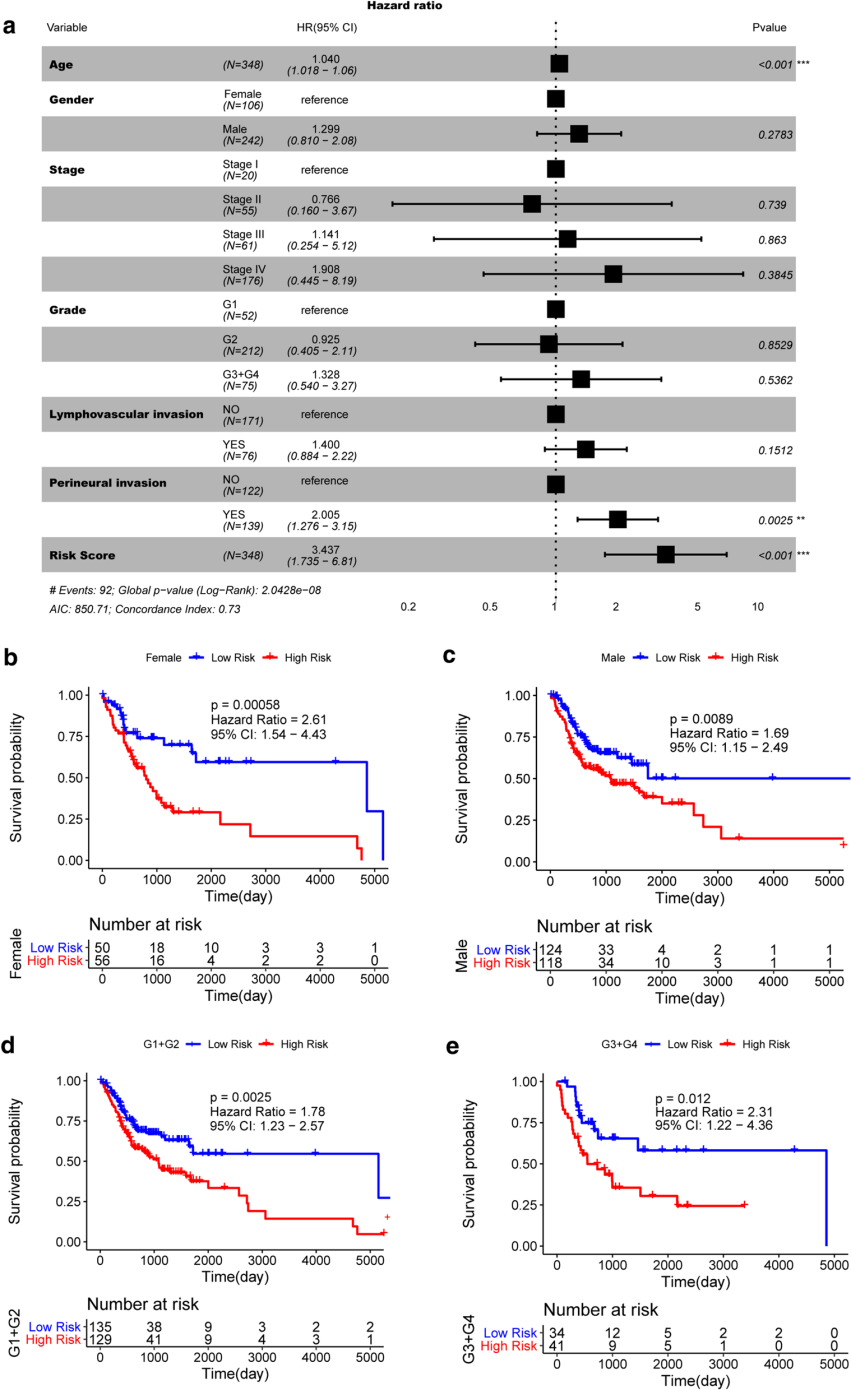
Risk Score is an independent marker of prognosis in OSCC. **a** The result of multivariate Cox regression analysis. Samples with Hazard ratio (HR) > 1 have a higher risk of death in comparison to the reference samples, and samples with HR < 1 have a lower risk of death. Kaplan Meier survival curves of OSCC samples with different clinicopathological factors, including female (**b**), male (**c**), Grade1 + Grade2 (D) and Grade3 + Grade4 (D). The horizontal axis represents time, the vertical axis represents survival rate, and different groups are represented by different colors. The P value is obtained from the log-rank test

We conducted multivariate Cox regression analysis, using the characteristic related to OSCC progression, including Age, Gender, TNM Stage, Grade, Lymphovascular invasion, Perineural invasion and Risk Score as variables to analyze whether Risk Score was an independent prognostic factor. It was found that the risk score is still significantly correlated with the OS, and the greater the risk of death with a higher Risk Score, further indicates that Risk Score was a factor for poor prognosis (HR = 3.437, 95%CI 1.735–6.81, P < 0.001). In addition, Age and Perineural invasion were also independent prognostic factors (Fig. 4a).

We regrouped OSCC patients by Gender and Grade and performed Kaplan–Meier survival analysis. Among the female samples (Fig. 4b), male samples (Fig. 4c), early grade (G1 + G2) samples (Fig. 4d), and late grade (G2 + G3) samples (Fig. 4e), the OS of the high-risk group was significantly worse than that in the low-risk group. These results suggested that the Risk Score can be used as an independent factor to predict the prognosis of OSCC patients.

### Nomogram model can predict the long-term OS of OSCC patients

**Fig. 5:**
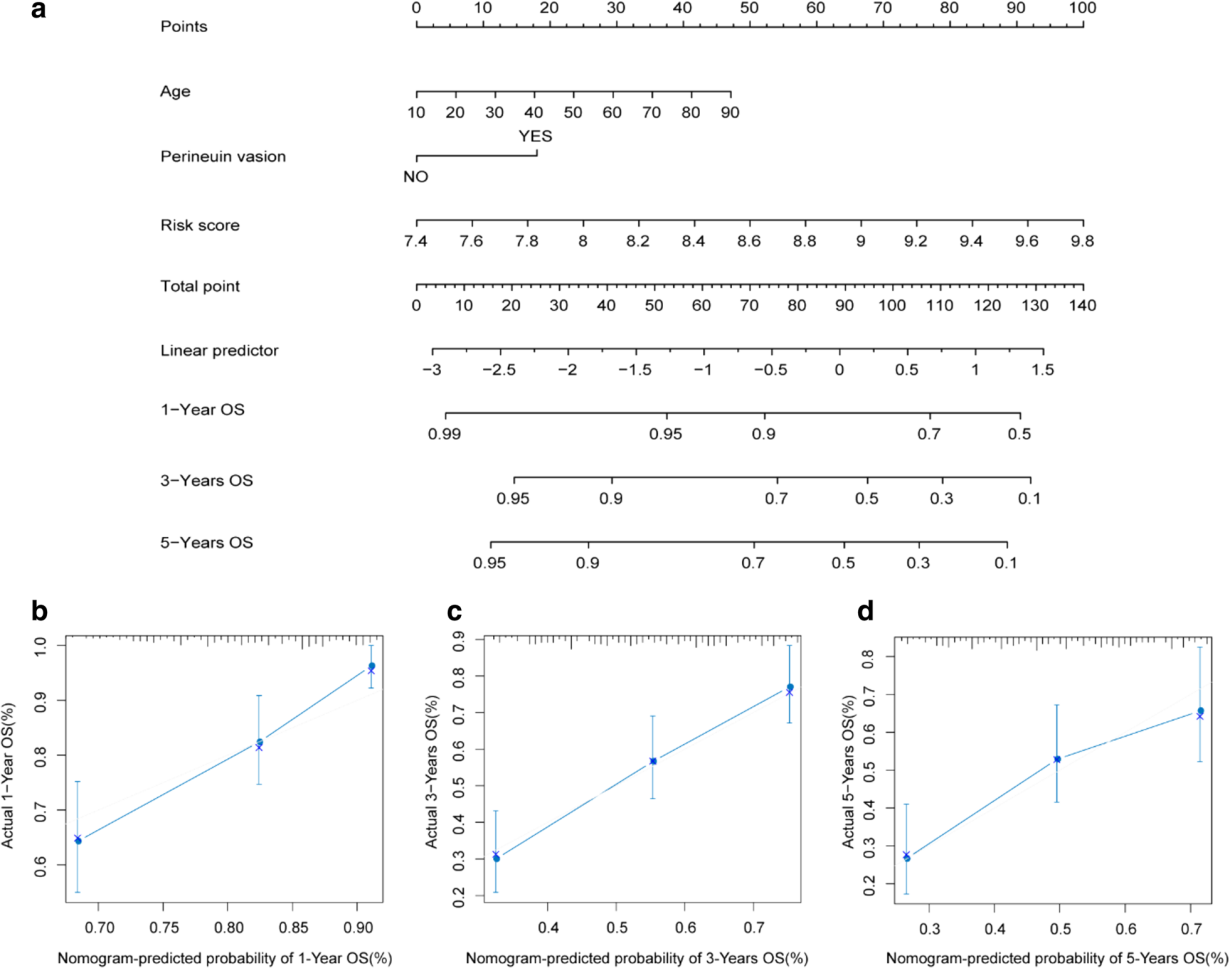
The nomogram for prediction of the survival probability of OSCC patient. **a** The nomogram for prediction of the 1-, 3-, and 5-year OS probabilities for OSCC patients. **b**–**d** The calibration curve for prediction of the 1- (**b**), 3- (**c**), and 5-year (**d**) OS probabilities for OSCC patients. The X axis denotes the survival rate predicted by Nomogram, and the Y axis denotes the actual survival rate, respectively

The Nomogram model was constructed based on the three independent prognostic factors, including Age, Perineural invasion and Risk Score (Fig. 5a). For each OSCC sample of TCGA, draw vertical lines upwards to determine the points obtained from each factor in the Nomogram. And the sum of these points is located on the "Total Points" axis, and then a line is drawn down from the Total Points axis to obtain the predicted OS probability of OSCC patients for 1, 3, and 5 years. And the calibration curve was close to the ideal curve in the calibration chart (grey straight line), indicating that the predicted OS probability was consistent with the actual (Fig. 5b–d). The Nomogram could effectively predict the long-term survival probability of OSCC.

## Discussion

OSSC originates from the oral cavity and oropharyngeal mucosa, representing one of the most common malignancies in the head and neck. In addition to causing local hyperplasia and surrounding tissue erosion, it often metastasizes to lymph nodes, seriously affecting the health and lives of patients [20]. we identified 4 hypoxia-related genes, which may related to the prognosis of OSCC patients.

Hypoxia is a key factor in tumor pathophysiology, causing changes in cell metabolism, triggering various molecular reactions, and promoting tumor spread. Hypoxia-inducible factors (HIFs) are the main molecules related to hypoxia research. It is known that HIFs regulate a large number of genes involved in cell proliferation, movement, metabolism, and angiogenesis through inducing the expression of its downstream target genes [21]. The process of epithelial to mesenchymal transition (EMT) is related to cancer metastasis. According to reports, hypoxia can cause EMT in many cancers, including breast, prostate and oral cancer [22]. Therefore, we speculated that hypoxia-related genes might have prognostic values in OSCC patients.

In this study, four hypoxia-related genes, ALDOA, P4HA1, PGK1 and VEGFA, were identified by LASSO Cox regression analysis, which were significantly associated with the overall survival rate of OSCC. Moreover, they were all risk genes for OSCC death event, i.e. their high expression would lead to poor prognosis. Human fructose diphosphate aldolase (ALDOA) and phosphoglycerate kinase 1 (PGK1) are the key rate-limiting enzymes in the process of aerobic glycolysis [23, 24]. Tumor cells tend to produce ATP through aerobic glycolysis rather than oxidative phosphorylation (Warburg Effect). In the hypoxic environment, Warburg Effect is induced by HIF-1 [25]. Therefore, key enzymes of glycolysis in the hypoxic microenvironment can be used as signal molecules to participate in the regulation of tumor-related signal pathways and enhance the migration or proliferation of tumors. Previously, studies have shown that ALDOA was highly expressed in colon cancer, pancreatic cancer and osteosarcoma [26–28], and was associated with their poor prognosis. PGK1 is directly regulated by HIF-1 in many tumors. Early studies have shown that PGK1 was activated by HIF-1 in colorectal cancer and liver cancer under hypoxia stress [29]. Due to HIF-1/PGK1-mediated epithelial-mesenchymal transformation (EMT), PGK1 is associated with the ability of cancer cells to metastasize [30], and the high expression of PGK1 is related to the inferior survival outcome of breast cancer patients [31]. Therefore, we speculated that ALDOA and PGK1 as key enzymes in the glycolysis process may be regulated by HIF-1, and the high expression of ALDOA and PGK1 in the hypoxic environment could enhance the proliferation and migration of tumors. Indeed, several researches has reported that ALDOA and PGK1 also mediated in OSCC and head and neck squamous cells carcinoma (HNSCC) [30, 32].Ahluwalia also reported that HIF-1 under hypoxic conditions regulates the expression of vascular endothelial growth factor A (VEGFA) at the transcriptional level [33]. The angiogenesis simulation (VM) [34] proposed by Maniotis in 1999 was a novel cancer marker, which has prognostic value in colorectal cancer, breast cancer, melanoma, etc. [35–37]. High VM expression was also considered to be a risk factor for poor prognosis, low survival rate, invasion and metastasis in patients with cancer, and HIF-1 expression was associated with VM [38]. In addition, the angiogenesis caused by hypoxia is usually VEGFA-dependent. This suggests that HIF regulation of VEGFA under hypoxic conditions may lead to high VM expression and a high risk in OSSC patients. Prolyl 4-hydroxylase subunit α1 (P4HA1) was encoding the active catalytic component of prolyl 4-hydroxylase (P4H), the key enzyme for collagen production [39]. It was reported that HIF-1 promotes P4HA1 expression to induce collagen deposition and a more aggressive phenotype in human breast cancer, leading to poor prognosis for breast cancer patients [40]. Perhaps the high level of P4HA1 expression decreases the OS probability of OSSC patients through the same process. These studies proved that our results were reliable and the over-expression of 4 genes might involve in OSCC progression and prognosis.

Immune infiltration has always been an important indicator of tumor prognosis [19]. Hypoxia was a prominent characteristic of chronically inflamed tissues, which could affect disease progression by inflammatory signaling pathways in immune and non-immune cells [41]. Thus, this study analyzed the differences in immune infiltration of 22 types of immune cells between OSCC patients in high and low risk groups, and found 7 types of immune cells: B cells naive, T cells CD8, T cells follicular helper, T cells regulatory (Tregs), Macrophages M0, Mast Cell activated and Neutrophils have significant differences in the degree of infiltration between high and low risk groups, and the infiltration proportion of immune cell in the high-risk group is higher, which indicated that immune infiltration is related to the prognosis of OSCC. The immune checkpoint expression has become a biomarker for cancer patients in the selection of immunotherapy. The study also found that the expression of the two immune checkpoints PDL2 and TIM3 were significantly different in the high and low risk groups. The expression in the high-risk group was significantly higher, indicating that patients in the high-risk group may more sensitive to the treatment of PDL2 and TIM3 inhibitor. Recently, combined targeting of TIM-3 and PD-1/PDL-1 has been proposed in hematological malignancies. It can be synergistically administered to control tumor growth [42]. It suggested that the poor prognosis of OSCC caused by hypoxia may be related to the immune microenvironment.

Overall, the prognostic model we constructed based on four optimal hypoxia-related genes that could effectively predict the survival probability of OSCC patients. However, the specific functions and potential mechanism of the above hypoxia-related genes in OSSC are required to be further explored.

## Supplementary Information

**Additional file 1: Table S1.** 26 hypoxia-related genes. **Table S2.** Analysis of correlations between risk score and immune checkpoints.

## Data Availability

The datasets of this study were downloaded from The Cancer Genome Atlas (TCGA, https://tcga-data.nci.nih.gov/tcga/) and the Gene Expression Omnibus (GEO, https://www.ncbi.nlm.nih.gov/geo/) database [GSE65858].
