# Supplementary material for: A four-hypoxia-genes-based prognostic signature for oral squamous cell carcinoma
Source: BMC Oral Health. 2021 May 3;21:232. doi: 10.1186/s12903-021-01587-z (PMC8094530; doi:10.1186/s12903-021-01587-z)
Supplement: Supplementary file 1 — Additional file 1: Table S1. 26 hypoxia-related genes. Table S2. Analysis of correlations between risk score and immune checkpoints. [file 12903_2021_1587_MOESM1_ESM.docx]

**A four-hypoxia-genes-based prognostic signature for Oral squamous cell carcinoma**

**Running title：A prognostic signature for OSCC**

Chenguang Zhao^1^, Yingrui Zhou^1^, Hongwei Ma^1^, Jinhui Wang^1^, Haoliang Guo^1^, Hao Liu^2^*,

1 Department of emergency and general dentistry, Tianjin Stomatology Hospital·Hospital of Stomatology, NanKai University·Tianjin Key Laboratory of Oral and Maxillofacial Function Reconstruction, Tianjin, 300041, China.

2 Department of Oral and Maxillofacial surgery, Tianjin Stomatology Hospital·Hospital of Stomatology, NanKai University·Tianjin Key Laboratory of Oral and Maxillofacial Function Reconstruction, Tianjin, 300041, China.

*Corresponding Author: Hao Liu

Department of Oral and Maxillofacial surgery, Tianjin Stomatology Hospital·Hospital of Stomatology, NanKai University·Tianjin Key Laboratory of Oral and Maxillofacial Function Reconstruction, No. 75, Dagu North Road, Heping District, Tianjin, 300041, China.

Email: haoliu_2020@outlook.com

haoliu@nankai.edu.cn

**Table S1 26 Hypoxia-related genes**

| **Rank** | **Hypoxia-related gene** |
| --- | --- |
| 1 | ALDOA |
| 2 | ANGPTL4 |
| 3 | ANLN |
| 4 | BNC1 |
| 5 | CA9 |
| 6 | CDKN3 |
| 7 | COL4A6 |
| 8 | DCBLD1 |
| 9 | ENO1 |
| 10 | FAM83B |
| 11 | FOSL1 |
| 12 | GNAI1 |
| 13 | HILPDA |
| 14 | KCTD11 |
| 15 | KRT17 |
| 16 | LDHA |
| 17 | MRGBP(C20orf20) |
| 18 | MRPS17 |
| 19 | P4HA1 |
| 20 | PGAM1 |
| 21 | PGK1 |
| 22 | SDC1 |
| 23 | SLC16A1 |
| 24 | SLC2A1 |
| 25 | TPI1 |
| 26 | VEGFA |

**Table S2 Analysis of correlations between risk score and immune checkpoints.**

| Variable 1 | Variable 2 | Pearson correlation coefficient | P value |
| --- | --- | --- | --- |
| Risk Score | CTLA4 | -0.073927468 | 0.168815221 |
| Risk Score | PDL1 | 0.051672386 | 0.336496905 |
| Risk Score | PDL2 | 0.136456675 | 0.010823745 |
| Risk Score | TIM3 | 0.109370857 | 0.041443358 |
| Risk Score | LAG3 | -0.048182432 | 0.370189601 |
| Risk Score | TIGIT | -0.055512359 | 0.301772304 |
| CTLA4 | PDL1 | 0.584371958 | 2.98E-33 |
| CTLA4 | PDL2 | 0.576210173 | 3.58E-32 |
| CTLA4 | TIM3 | 0.769189943 | 2.84E-69 |
| CTLA4 | LAG3 | 0.756930559 | 6.7E-66 |
| CTLA4 | TIGIT | 0.862190533 | 3.2E-104 |
| PDL1 | PDL2 | 0.816422438 | 1.6E-84 |
| PDL1 | TIM3 | 0.583633943 | 3.74E-33 |
| PDL1 | LAG3 | 0.597751996 | 4.34E-35 |
| PDL1 | TIGIT | 0.614316592 | 1.74E-37 |
| PDL2 | TIM3 | 0.643808606 | 4.03E-42 |
| PDL2 | LAG3 | 0.547688657 | 1.25E-28 |
| PDL2 | TIGIT | 0.556660992 | 1.04E-29 |
| TIM3 | LAG3 | 0.764162646 | 7.26E-68 |
| TIM3 | TIGIT | 0.824574464 | 1.33E-87 |
| LAG3 | TIGIT | 0.813609298 | 1.71E-83 |
